# Upper gastrointestinal hemorrhage caused by cholecystolithiasis compressing the cystic artery: a case report

**DOI:** 10.3389/fsurg.2026.1816881

**Published:** 2026-04-30

**Authors:** Xiaoming Zhou, Zhu Li

**Affiliations:** 1 Clinical Medical College, Guizhou Medical University, Guiyang, Guizhou, China; 2 Department of Hepatobiliary Surgery, The Affiliated Hospital of Guizhou Medical University, Guiyang, Guizhou, China

**Keywords:** angiography, case report, cholecystolithiasis, endoscopy, gastrointestinal hemorrhage, hemobilia, laparotomy

## Abstract

The majority of patients with cholecystolithiasis are asymptomatic. Hemobilia is a rare complication of cholecystolithiasis. It is also an uncommon cause of upper gastrointestinal hemorrhage, where the source of bleeding may be difficult to diagnose. This case report describes a middle-aged woman who experienced recurrent upper gastrointestinal hemorrhage both in the pre-admission period and during hospitalization. Treatment with medication, endoscopy, and interventional procedures yielded poor results, and the cause of the upper gastrointestinal hemorrhage was difficult to confirm. Ultimately, the diagnosis was confirmed and the condition was treated through exploratory laparotomy. In this report, we analyze the diagnostic and treatment process in this case to improve clinicians’ understanding of the causes of upper gastrointestinal hemorrhage, the complications of cholecystolithiasis, and the surgical indications for cholecystolithiasis or upper gastrointestinal hemorrhage.

## 1 Introduction

Upper gastrointestinal hemorrhage is a common clinical condition with multiple etiologies. Cholecystolithiasis is also a frequent clinical disorder, with complications primarily including acute cholecystitis, acute cholangitis, gallstones, and pancreatitis (1). Hemobilia is an uncommon complication of cholecystolithiasis. A direct injury to the cystic artery and a subsequent rupture with bleeding caused by cholecystolithiasis are extremely rare in clinical practice. It is difficult to make a definitive diagnosis in this situation through routine examinations, which can easily delay diagnosis and treatment (2–4). This report presents a rare case of upper gastrointestinal hemorrhage that was confirmed by surgical exploration to be caused by cholecystolithiasis compressing the cystic artery, leading to hemobilia. The patient's initial symptom was hematemesis. Physical examination only revealed signs of anemia and cholecystitis, and subsequent laboratory tests indicated progressively worsening anemia. The patient ultimately survived due to surgical intervention.

## 2 Case presentation

**Figure 1:**
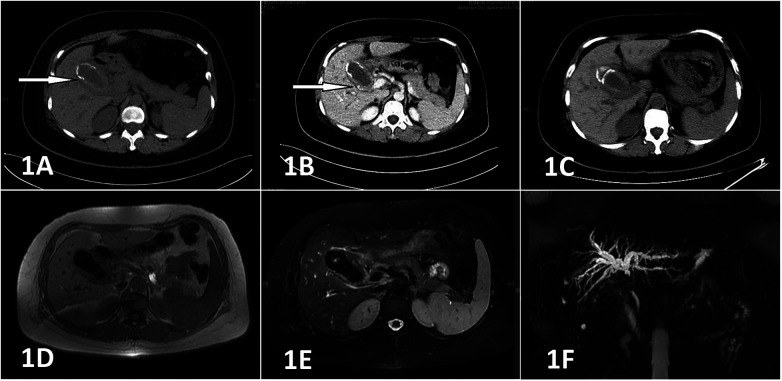
Initial plain CT scan shows an enlarged gallbladder with thickened walls and multiple ring-like hyperdense lesions within [**(A)**, ↑]. Contrast-enhanced CT demonstrates heterogeneous wall enhancement [**(B)**, ↑]. Follow-up plain CT scan shows worsened blurring of the surrounding fatty margins **(C)**. Plain MRI scan with T1-weighted imaging reveals a thickened gallbladder wall with low signal intensity within **(D)**; T2-weighted imaging demonstrates a gallbladder filled with short T2 signal intensity and the thickened neck wall contains cord-like low signal intensity **(E)**. MRCP shows intrahepatic biliary dilatation with abrupt narrowing of the common bile duct, suggestive of Mirizzi syndrome **(F)**.

**Figure 2:**
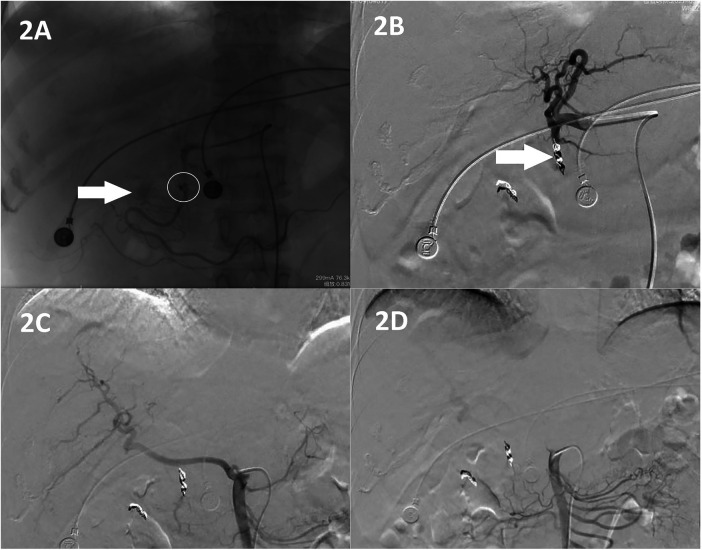
The gastrointestinal angiography shows contrast extravasation in the gastroduodenal artery and its branches [**(A)**, O] with partial hazy diffusion [**(A)**, ↑]. Therefore, gastroduodenal artery embolization is performed [**(B)**, ↑]. In addition, there is no significant contrast extravasation in the proper hepatic artery **(C)** or its branches, including the cystic artery. The superior mesenteric artery **(D)** and its branches also show no extravasation.

A 46-year-old female patient was admitted to our hospital with a 15-year history of cholecystolithiasis and 14 days of hematemesis. The cholecystolithiasis was detected during a health examination 15 years prior. Three months before admittance, she experienced right upper quadrant pain and 14 days before admittance, she presented to another hospital with hematemesis. Both the computed tomography (CT) and magnetic resonance imaging (MRI) scans indicated acute calculous cholecystitis, with the MRI scan additionally revealing intrahepatic biliary dilatation with an abrupt narrowing of the common bile duct, suggestive of Mirizzi syndrome (Figure 1). Endoscopy at this hospital showed ulcerative lesions at the duodenal bulb. Consequently, an interventional angiographic gastroduodenal artery embolization procedure was performed, after which the hematemesis was temporarily controlled (Figure 2).

**Figure 3:**
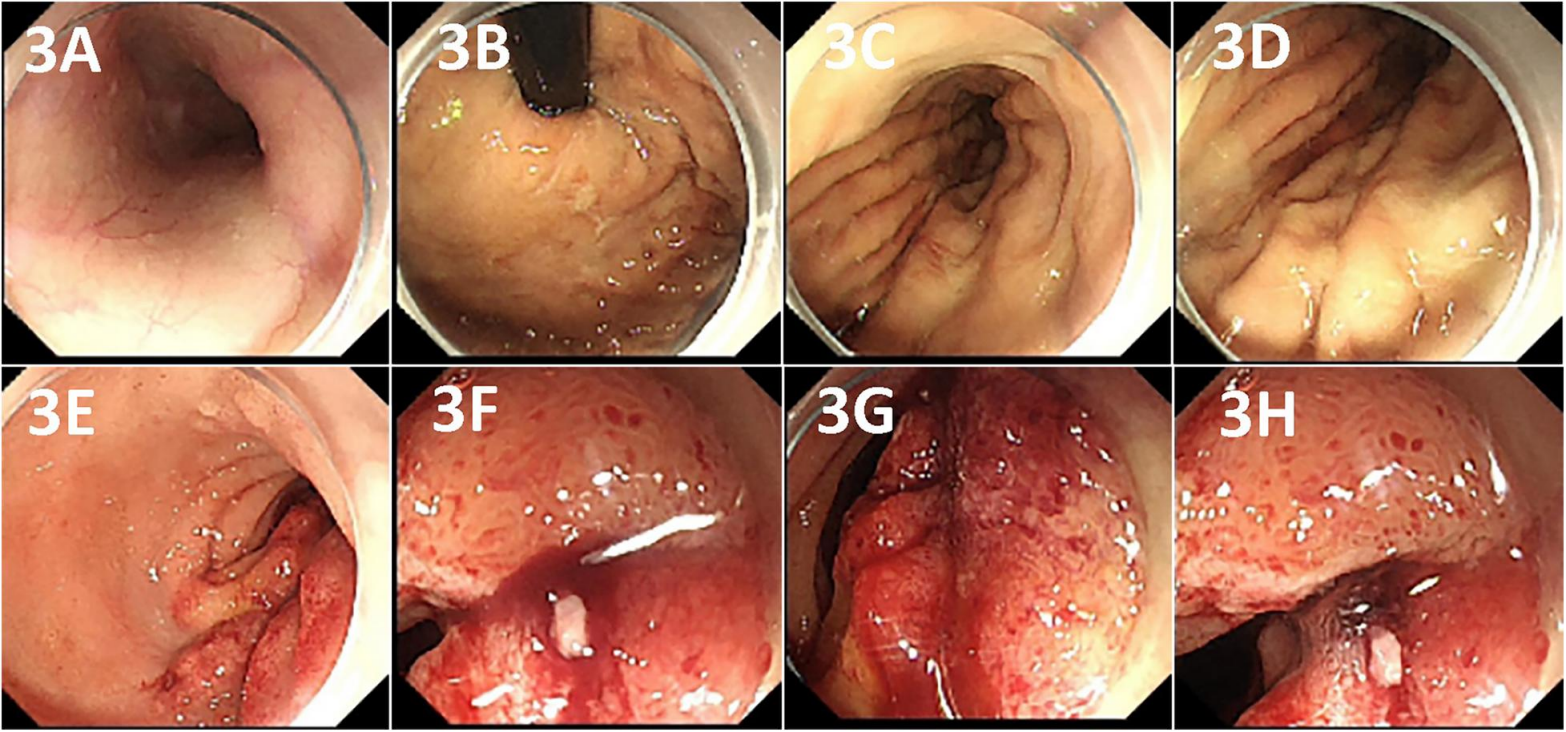
Endoscopy shows no significant lesions in the esophagus **(A)** or stomach **(B–D)**. The bleeding is visible at the posterior wall of the duodenal bulb (**E–G),** and hemostasis is achieved by applying titanium clips **(H)**.

The patient presented to our hospital for the treatment of cholecystolithiasis. There were no significant medical, personal, or family histories. A physical examination revealed a temperature of 36.9 °C, anemic appearance, flat abdomen, and tenderness in the right upper quadrant, and was positive for Murphy's sign (+); there were no other significant findings. The laboratory test results were as follows: white blood cell count, 9.07 × 10⁹/L; red blood cell (RBC) count, 2.25 × 10^12^/L; hemoglobin (Hb), 65.00 g/L; hematocrit (Hct), 19.10%; prothrombin time, 14.00 s; and activated partial thromboplastin time, 34.80 s. After admission to our hospital, the patient presented with hematemesis (approximately 350 mL) and melena (approximately 300 mL). The treatment included fasting, administration of a proton pump inhibitor and anti-infective agents, and a blood transfusion. The gastrointestinal bleeding was suspected to originate from the duodenum. Therefore, we conducted a repeat endoscopy, which revealed bleeding from the duodenal bulb that was treated with hemostasis using titanium clips (Figure 3). However, the patient experienced intermittent hematemesis and melena following the endoscopic hemostasis treatment.

On the second day of admission, the follow-up test results were as follows: Hb, 56.00 g/L; Hct, 17.20%. During the blood transfusion, the patient presented with 300 mL of hematemesis and 100 mL of melena. As the cause of the active gastrointestinal bleeding remained unclear, an emergency exploratory laparotomy was performed.

The intraoperative findings are as follows. There was no hemoperitoneum. We examined the stomach and the duodenal bulb without finding any significant bleeding. The fundus of the gallbladder was then incised, and we found multiple large stones (approximately 3 cm) and copious clotted blood within the gallbladder. After removing three stones, one stone remained firmly lodged adjacent to the neck of the gallbladder. When we attempted to dislodge it slightly, copious blood surged from the gallbladder. A porta hepatis blockage belt was applied to occlude the blood vessels at the hepatic portal; this markedly reduced the hemorrhage, after which the stone at the gallbladder neck was removed. Subsequently, we identified a vascular structure approximately 3 mm in diameter within the submucosal layer of the gallbladder neck. This vessel exhibited a longitudinal tear measuring approximately 2 mm in length. After we sutured the vascular tear, the bleeding stopped. The gallbladder cavity was then thoroughly irrigated and debrided, revealing extensive clotted blood and necrotic gallbladder mucosa. The debrided necrotic tissue was sent for a routine histopathological examination. At this point, the patient had lost 2,500 mL of blood and developed hypotension. To save the patient's life, it was decided to abandon the cholecystectomy and instead perform a cholecystostomy. We confirmed that there was no other bleeding within the abdominal cavity. Finally, we placed two drainage tubes and performed a layered closure of the abdominal wall.

After the operation, the patient was transferred to the intensive care unit for advanced life support. On postoperative day 3, the laboratory test results were as follows: RBC count, 3.34 × 10^12^/L; Hb, 97.00 g/L; Hct, 28.40%. Two drainage tubes were removed on postoperative days 10 and 11, respectively. The pathology results confirmed acute gangrenous cholecystitis with hemorrhage. The patient was discharged successfully on postoperative day 12. During the subsequent 6-month follow-up period, the patient's condition remained favorable.

## 3 Discussion

The European Guidelines on Cholelithiasis indicate that approximately 80% of individuals with cholecystolithiasis exhibit no associated symptoms (5). The progression of cholecystolithiasis generally unfolds in the following three phases: the asymptomatic, symptomatic, and complicated stages (3). The complication rate for asymptomatic cholecystolithiasis is extremely low, with an annual incidence of merely 0.1%–0.3% (5, 6). For patients with asymptomatic cholecystolithiasis, the efficacy of cholecystectomy remains uncertain and routine cholecystectomy is not recommended (5, 6). However, cholecystectomy is still advised if typical symptoms develop or high-risk factors for gallbladder carcinoma are present (5, 6). Therefore, patients with asymptomatic cholecystolithiasis should be informed of the possible symptoms and risk of complications. Close follow-up should guide patients to undergo regular relevant examinations to monitor their clinical condition.

Hemobilia is defined as bleeding into the biliary tree. The first recorded case appears to have been reported in 1654 by Francis Glisson (7). Symptomatic hemobilia typically manifests as biliary colic, jaundice, and upper gastrointestinal bleeding, named Quincke's triad (4, 7, 8). However, only approximately 22%–35% of patients with hemobilia present with the classic Quincke's triad. This makes hemobilia an important consideration in the differential diagnosis of upper gastrointestinal hemorrhage (2, 4, 7, 8). Cholelithiasis accounts for approximately 5%–15% of the etiological composition of hemobilia (7). Massive upper gastrointestinal hemorrhage directly caused by cholecystolithiasis is extremely rare in clinical practice. Bockova et al. reported a case of upper gastrointestinal hemorrhage caused by gallbladder mucosal erosion and hemorrhage resulting from calculous cholecystitis, which was ultimately cured by cholecystectomy (9). Chang et al. reported a case of mild hemobilia caused by choledocholithiasis, which was diagnosed via endoscopic retrograde cholangiopancreatography (ERCP) (10). The presumed mechanism of cystic artery rupture and bleeding in this case was sustained mechanical compression of the cystic artery by a large impacted stone at the gallbladder neck, resulting in chronic local vascular wall erosion and ultimately a longitudinal tear that led to hemobilia. Pseudoaneurysm formation was also a possible mechanism; although an unrecognized pseudoaneurysm cannot be completely excluded as an intermediate process, mechanical erosion was considered the primary mechanism in this case (11). To date, no case reports exist of cholecystolithiasis compressing the cystic artery, leading to a massive upper gastrointestinal hemorrhage. Consequently, clinicians should consider the possibility of this potentially fatal condition and take it into account when making a diagnosis. In addition to conducting routine examinations such as ultrasound, CT, MRI, and endoscopy, if the patient's hemodynamics are stable, ERCP, magnetic resonance cholangiopancreatography, computed tomography angiography, angiography, and other tests should be conducted to confirm the diagnosis.

Upper gastrointestinal hemorrhage is a common clinical condition with varied etiologies. The most frequent cause is peptic ulcer disease, accounting for approximately 40%–50% of the cases (12–14). Numerous studies indicate that hemobilia represents a rare cause of upper gastrointestinal hemorrhage (2, 13). In this case, the endoscopy results supported a diagnosis of a duodenal ulcer as the source of the patient's hemorrhage. However, the patient experienced substantial blood loss and recurrent active gastrointestinal hemorrhage despite management with medication, interventional arterial embolization, and endoscopic treatment. This indicates that endoscopy may yield misdiagnoses or missed diagnoses in some complex upper gastrointestinal hemorrhage scenarios. The etiology should be meticulously evaluated by integrating the patient's medical history, clinical signs, and auxiliary examinations; endoscopy alone should not be regarded as the sole definitive standard. Furthermore, for upper gastrointestinal hemorrhage where the diagnosis remains unclear, if the patient continues to experience active bleeding despite aggressive treatment, prompt surgical exploration should be undertaken to identify the bleeding site and cause, which helps prevent delays in diagnosis and treatment.

## 4 Conclusion

In summary, when patients present with upper gastrointestinal hemorrhage and have a history of cholecystolithiasis, the possibility of the bleeding originating from the gallbladder should be considered. If the diagnosis remains unclear after a thorough examination, and when medical, interventional, and endoscopic treatments do not reduce active bleeding that is compromising the patient’s vital signs, surgical exploration should be decisively performed to identify the source of bleeding and prevent further harm to the patient's health.

## Data Availability

The original contributions presented in the study are included in the article/supplementary material, further inquiries can be directed to the corresponding author.
